# A mixed community of skin microbiome representatives influences cutaneous processes more than individual members

**DOI:** 10.1186/s40168-020-00963-1

**Published:** 2021-01-22

**Authors:** Kristin H. Loomis, Susan K. Wu, Amanda Ernlund, Kristina Zudock, Allison Reno, Kianna Blount, David K. Karig

**Affiliations:** 1 Research and Exploratory Development, Johns Hopkins University Applied Physics Laboratory, Laurel, MD USA; 2 Department of Bioengineering, Clemson University, Clemson, SC USA

**Keywords:** Skin microbiome, Skin proliferation, Host-microbiome interaction, RNA sequencing, Cutaneous transcriptome, Human microbiome, Human skin equivalents

## Abstract

**Background:**

Skin, the largest organ of the human body by weight, hosts a diversity of microorganisms that can influence health. The microbial residents of the skin are now appreciated for their roles in host immune interactions, wound healing, colonization resistance, and various skin disorders. Still, much remains to be discovered in terms of the host pathways influenced by skin microorganisms, as well as the higher-level skin properties impacted through these microbe-host interactions. Towards this direction, recent efforts using mouse models point to pronounced changes in the transcriptional profiles of the skin in response to the presence of a microbial community. However, there is a need to quantify the roles of microorganisms at both the individual and community-level in healthy human skin. In this study, we utilize human skin equivalents to study the effects of individual taxa and a microbial community in a precisely controlled context. Through transcriptomics analysis, we identify key genes and pathways influenced by skin microbes, and we also characterize higher-level impacts on skin processes and properties through histological analyses.

**Results:**

The presence of a microbiome on a 3D skin tissue model led to significantly altered patterns of gene expression, influencing genes involved in the regulation of apoptosis, proliferation, and the extracellular matrix (among others). Moreover, microbiome treatment influenced the thickness of the epidermal layer, reduced the number of actively proliferating cells, and increased filaggrin expression. Many of these findings were evident upon treatment with the mixed community, but either not detected or less pronounced in treatments by single microorganisms, underscoring the impact that a diverse skin microbiome has on the host.

**Conclusions:**

This work contributes to the understanding of how microbiome constituents individually and collectively influence human skin processes and properties. The results show that, while it is important to understand the effect of individual microbes on the host, a full community of microbes has unique and pronounced effects on the skin. Thus, in its impacts on the host, the skin microbiome is more than the sum of its parts.

Video abstract.

**Supplementary Information:**

The online version contains supplementary material available at 10.1186/s40168-020-00963-1.

## Background

Human skin provides a physical barrier between the body and the outside world, preventing the entry of irritants and pathogens, informing the development of immune responses, and regulating water loss [1–4]. Populations of microorganisms—the skin microbiome—reside on and within human skin. Different skin sites and individuals harbor varying compositions of microorganisms [5, 6] with estimated densities ranging from 10^4^ to 10^6^ microorganisms per square cm [6].

Metagenomics sequencing studies have explored the composition and characteristics of the skin microbiome. While bacteria dominate the microbiome composition [7], viruses, fungi, and even mites have also been identified [4]. The bacterial microbiome primarily consists of four phyla—Actinobacteria, Firmicutes, and Proteobacteria—and a lower abundance of Bacteroidetes [5]. Yet, the composition of particular taxa varies widely across body sites. Areas such as the antecubital fossa (inner elbow) or the axillary vault (underarm) are classified as moist regions and are associated with a high abundance of *Staphylococcus* and *Corynebacterium* [4]. Drier areas, such as the forearm and legs, harbor more diverse populations of bacteria. One study found that over half of the bacterial genera at dry sites can be attributed to either *Cutibacterium* (recently renamed from *Propionibacterium* [8]), *Corynebacteria*, *Staphylococcus*, *Streptococcus, or Acinetobacter* species [4, 9]. Sebaceous areas, such as the forehead or alar crease (side of the nostril) harbor *Cutibacterium*, which primarily resides within sebaceous glands [4]. Culture-based studies have additionally characterized the skin microbiome while also providing microbial isolates for experimental studies [9–12]. Additionally, culture-based studies recognize bacteria that are viable on the skin as well as those that may have been underestimated due to sequencing biases [13, 14]. These studies have shown that *Cutibacterium*, *Staphylococcus*, *Micrococcus, Bacillus, Roseomonas,* and *Paenibacillus* are prevalent and viable on the skin microbiome [10].

The skin microbiome plays a role in directing cutaneous processes critical to human health and disease [7, 15–18]. Many previous research efforts have detailed specific mechanisms of communication between commensal skin microorganisms and host tissue. For example, *Staphylococcus epidermidis* and *Staphylococcus aureus* have been found to induce distinct signaling pathways, leading to specialized modulation of the innate immune system [19]. Similarly, a cell wall component common to the *Corynebacterium* genus was found to modulate an additional distinct pathway of the immune system, interleukin-23 (IL-23)-dependent inflammation [20]. In disease states, abnormal microbiome compositions—often characterized by a reduced diversity of microorganisms—have also been linked to diabetes, psoriasis [21–23], and atopic dermatitis [24–28].

While a wealth of discoveries has been made regarding the impact of skin microorganisms on the host, the vast majority of efforts have focused on individual taxa, have concentrated on specific impacts on the skin, or have drawn conclusions statistically from human sampling studies. Much remains to be discovered in terms of the collective host pathways that skin microbes influence at the gene expression level, as well as the higher-level skin properties impacted through the modulation of these pathways.

Towards this end, germ-free mice (those reared without a microbiome) have offered a powerful tool. Analogous to studies in the gut, where the microbiome has been shown to modulate fundamental functions such as intestinal nutrient absorption and mucosal barrier fortification [29–31], studies in the skin have demonstrated microbiome modulation of wound healing [32] and epidermal differentiation [33]. Of particular relevance, Meisel et al. revealed that in healthy mice, the skin microbiome influences gene expression for a range of biological processes including the cutaneous immune response, cytokine production, epidermal differentiation, and epidermal development [33]. Despite these significant findings, additional efforts are needed to characterize the consummate influence of individual microbiome members on microbiome-host dialogue. In addition, the uniqueness of the human skin microbiome warrants the characterization of microbe-host interactions in human tissues [34, 35].

Here, we examine how members of the human skin microbiota inform cutaneous processes when cultured both individually and in a mixed community. To accomplish this, we use microbial isolates from healthy human skin and three-dimensional human skin equivalents. The human skin equivalents, like germ-free mouse models, allow for carefully controlled studies of skin-microorganism interactions, [36] but they additionally support the study of human-specific tissues and microorganisms [37–41]*.* We studied microbiome representatives from species and genera that commonly reside in the aerobic environments of the skin surface—*Staphylococcus epidermidis*, *Streptococcus luteciae*, *Bacillus* sp., *Roseomonas mucosa*, *Paenibacillus* sp., *Micrococcus luteus, Corynebacterium* sp., and *Acinetobacter lwoffi* [5]*.* We investigate individual microorganism contributions to a collective community response by co-culturing individual microorganisms and a mixed community at the air-tissue interface.

Using transcriptomics and histological analyses, we find that the presence of a model microbiome leads to significantly altered patterns of gene expression, influencing genes involved in the regulation of apoptosis, proliferation, and the extracellular matrix. Moreover, microbiome treatment influences the thickness of the epidermal layer, reduces the number of actively proliferating cells, and increases filaggrin expression. Many of these findings are evident upon treatment with the mixed community, but not detected in treatments by single microorganisms, underscoring the impact of a diverse microbiome on the skin-microbiome relationship. This work furthers our understanding of how microbiome constituents both individually and collectively influence skin processes and extends previous efforts in murine systems [33] to human tissue and relevant microorganisms.

## Methods

### Skin microbiome representatives

**Fig. 1:**
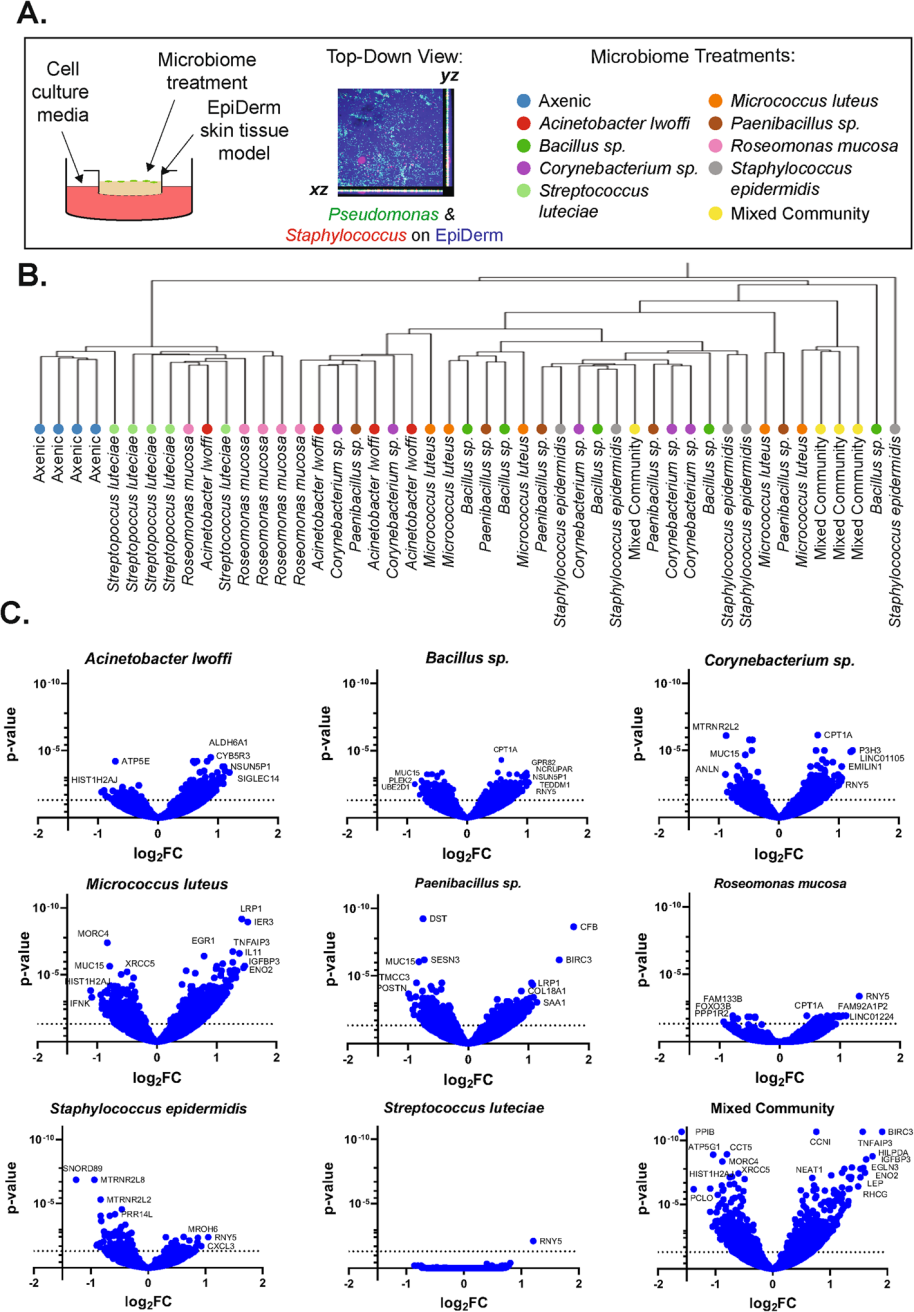
Global view of transcriptomics data. **a** Experimental overview showing (left) a schematic of the 3D skin tissue culture system, (middle) a confocal image of EpiDerm skin tissue co-cultured with fluorescently labeled bacteria, and (right) all microbiome treatments included in this study. **b** Hierarchical clustering plot of replicate treatment conditions. **c** Volcano plots showing log2FC and adjusted *p* values for genes of the indicated treatment condition compared to the axenic control

Bacteria used in this study were isolated from swabs of healthy human skin at either the forearm, antecubital fossa, or forehead. Details on the bacterial isolations are further described by Timm et al. [42]. The V1 to V9 regions of the 16S ribosomal RNA (rRNA) gene were obtained by Sanger sequencing (Genewiz, LLC). Forward and reverse reads were merged to form a consensus sequence, which was then classified using the SINA search and classify service against the small subunit references in the Greengenes, RDP, and SILVA databases [43]. The classification based on the Greengenes database was used to identify isolates. Table S1 shows isolate IDs, 16S rRNA consensus sequences, classification results, and the skin site of isolate collection, as provided by Timm et al. [42]. For preliminary microscopy studies (shown in Figures 1a and S2), fluorescent reporter strains of *Staphylococcus aureus* and *Pseudomonas aeruginosa* were used. Specifically, *Staphylococcus aureus* RN4220 was transformed with pAH9, which codes for expression of the fluorescent protein mCherry [44] and was a gift from Alexander R. Horswill. *Pseudomonas aeruginosa* PAO1 was transformed with pMRP9-1, enabling expression of green fluorescent protein, and was a gift from Pete Greenberg.

### Bacteria culturing

Bacteria were stored at – 80 °C in tryptic soy broth (TSB, Sigma Aldrich) supplemented with 10% glycerol. Bacteria were streaked on tryptic soy agar (TSA, Hardy Diagnostics) at room temperature until single colonies were visible. Individual colonies were then picked, inoculated into 3 ml of TSB, and cultured at 30 °C overnight. Overnight cultures were then diluted 1:500 and incubated for 4 h to generate starter inoculation cultures. *Corynebacterium* sp. and *Streptococcus luteciae*, which grow more slowly than other bacteria used in this study, were cultured on TSA for up to 4 days, in the initial liquid culture for 72 h, and then in the subsequent liquid culture for 18 h.

### OD and CFU/ml calibration curves

Starter inoculation cultures were used to generate seven cultures at dilutions ranging from 1:40 to 1:4 × 10^6^ in TSB. Bacterial cultures were allowed to grow from 4 to 18 h, and the optical density at 600 nm (OD) was measured using the cuvette reading mode on a Nanodrop 2000C spectrophotometer (Thermo Scientific). For cultures where the OD reading was between 0.1 and 1, bacteria were serially diluted from 1:100 to 1:10^6^ and plated in triplicate on TSA plates. The number of colony-forming units (CFU) was counted for each condition, and a linear regression was calculated using GraphPad Prism (version 8.1.1 for windows, GraphPad Software, La Jolla, CA, USA, www.graphpad.com) to relate CFU/mL to the culture OD. These relationships are shown in Figure S1.

### EpiDerm and bacteria co-culture

Underdeveloped full-thickness EpiDerm (EFT-400-7A, MatTek) was equilibrated and cultured in antibiotic-free culture media (MatTek) at 37 °C with 5% CO_2_ and no humidification. The tissues were cultured by MatTek for 2 days without antibiotics prior to shipment and then cultured in the antibiotic-free cell culture medium (provided with EFT-400-7A by request, MatTek), which was changed every other day. We assume that due to regular media changes and buffering of the media (HEPES), pH changes due to evaporation are negligible. The media also contains a pH indicator (phenol red), and no problematic observations were made during the course of the experiments. Starter inoculation cultures were washed twice in phosphate buffered saline (PBS, Fisher BioReagents) by centrifugation at 8000×*g*, removal of the supernatant, and suspension of the cell pellet in fresh PBS. The cell concentration was then adjusted to 1 × 10^8^ CFU/ml based on the established OD vs. CFU/ml relationships. For the mixed community treatment, equal volumes of cell suspensions were mixed to generate a total suspension of 1 × 10^8^ CFU/ml. Once the EpiDerm tissues were fully developed (after 3 days of culture upon receipt), bacteria solutions were deposited at the air-tissue interface (see Fig. 1a and S2) in two spots of 2.5 μl each and allowed to dry. The axenic control condition was treated with the PBS vehicle only. For transcriptomics analysis, five tissues were used for each condition, and bacteria were incubated on the tissue for approximately 18 h. For histological analysis, five to six tissues were used per condition, and bacteria were incubated on the tissues for 5 days.

### RNA extraction and sequencing

To extract cellular RNA, entire tissues were immersed in Trizol and lysed by bead beating for five minutes at a 20 s^−1^ frequency using the Qiagen Tissue Lyzer with the Navy bead beating kit (Next Advance). Tissues homogenates were further lysed by passing through the Qiashredder (Qiagen). Homogenates were then extracted with chloroform, mixed with an equal volume of ethanol, and loaded into columns of an RNeasy mini kit (Qiagen), at which point the manufacturer’s directions were followed, which included the on-column DNAse (Qiagen) digestion. RNA concentrations were determined using a Quant-it RNA Assay Kit with a Qubit 3.0 Fluorometer. Library preparation was performed using the QuantSeq 3′ mRNA-Seq kit (Lexogen), which using oligo-dT priming and does not require the use of poly(A) enrichment or rRNA depletion [45]. Libraries were sequenced on a NextSeq using the 500/550 High Output v2 kit (Illumina) in 75 base pair single-read mode.

### Sequence processing and analysis

Raw single-end FASTQ files were trimmed with Trimmomatic-0.35 and adapters were removed using default settings [46]. Both leading and trailing minimum quality scores were set to 20, a sliding window of 4:20 was used, and the minimum read length was set to 50. Transcript expression was quantified with *Salmon* [47] and transcript level abundance was summarized by gene-level analysis using the tximport package in R [48]. The human genome assembly hg38 (NCBI assembly ID 5800238) was used as the index and bootstrapping (with replacement) was set at 50. Differential abundance was calculated using DESeq2 [49] with a false discovery rate (FDR) threshold of 0.1 [50]. DESeq2 uses the Benjamin-Hochberg (BH) correction to calculate adjusted p-values. Quality measures were examined, such as those pertaining to transcriptome coverage, and are shown in Table S3. A Pearson correlation was also performed to determine the similarity of the five biological replicates for each condition (Table S3). Samples with low Pearson correlations (< 0.875) to replicates were considered outliers [51] and removed (see Table S3). These outlier samples also exhibited more variable quality control metrics, such as lower coverage of the transcriptome. The differential expression analysis was then repeated for each condition. An adjusted *p* value of 0.05 was used as a significance cutoff to determine differentially expressed genes. To examine bacterial RNA presence, reads were classified at the genus-level using Kraken2 with a confidence-value of 0.2 [52]. Analysis of bacterial reads is shown in Figure S3.

To examine microbial expression from genes encoding for antimicrobial proteins and peptides (AMPs), a literature search was conducted to find common AMP-encoding genes in skin tissue. Euler diagrams were created with eulerAPE v3 [53]. Hierarchical clustering was performed using variance stabilizing transformed (VST) data for individual samples (as shown in Fig. 1b) [49] or log2 fold change (log2FC) values (as shown elsewhere throughout the paper) for aggregated treatment groups using average linkage in JMP® (Version 13.0.0, SAS Institute Inc., Cary, NC, 1989-2019) software. To examine expression levels of individual genes across tissues, VST-transformed data were plotted for individual tissues in a given condition.

### Gene overrepresentation analysis

To gain insight into the biological processes influenced by microbiome treatment, genes that were differentially expressed between the mixed community treatment and axenic control were analyzed with Protein ANalysis THrough Evolutionary Relationships (PANTHER) analysis tools [54]. First, the number of genes differentially expressed between the mixed community treatment and the axenic control were functionally classified to the PANTHER GO-Slim ontologies of Molecular Function, Biological Function, and Cellular Component. The annotation was repeated using a list of genes that were differentially expressed in both the mixed community and single microorganism treatment. Next, the PANTHER classification system [54] was used to conduct an overrepresentation analysis (Released 2019-07-11) using the GO Biological Processes Complete annotation (version 14.1, released 2019-07-03) [55, 56]. The input gene lists were based on all genes differentially expressed between the mixed community and the axenic control with a BH-adjusted *p* value < 0.05 (Table S4). Genes that were differentially expressed in the mixed community treatment condition but not in any single microorganism treatment were also examined using a BH-adjusted *p* value of < 0.05. A background list (included in Table S5) consisted of all genes detected across all samples in the experiment with a base mean over 1. To determine statistically overrepresented gene sets, the Fisher’s exact type test with a FDR correction was used. Reduce and Visualize Gene Ontology (REViGO) [57] was used to summarize the list of gene sets and find representative subsets. For analyses of the mixed community, the list of gene sets with a FDR *p* value< 0.01 were input into REViGO using the *Homo sapiens* database, the Resnik (normalized) similarity measure, and the option to allow for medium similarity. The resulting treemap was generated using *p* values to determine box size and was visualized using JMP statistical software. The REViGO treemap for the gene sets enriched in the mixed community but not single-microorganism treatments, gene sets with an FDR *p* value < 0.05 was used.

### Histology and immunofluorescence staining

EpiDerm tissues were removed from transwell inserts using a sterile scalpel and immediately incubated in 4% paraformaldehyde diluted in PBS. The Johns Hopkins University Reference Histology Center then embedded tissues in paraffin and obtained 5-μm-thick sections. Tissue sections were acquired approximately one-quarter of the way through the tissue and were collected approximately 25 μm apart. Tissues were deparaffinized and rehydrated by treatment in xylene and ethanol and then stained with hematoxylin and eosin (H&E). For immunofluorescence analysis, after deparaffinization, slides were submerged in citric acid antigen retrieval solution (BD Biosciences) under steam treatment for 20 min. Tissues were allowed to cool at room temperature for 30 min, were washed three times in PBS for 5 min, and were then permeabilized by incubating for 15 min in 0.1% triton-X 100 in PBS. Sections were then blocked by incubation in 5% bovine serum albumin (BSA) in PBS for 30 min at 37 °C, stained with a primary antibody overnight at 4 °C in a humidification chamber, washed three times in PBS for 2 min each, and then incubated with 1:200 dilutions of secondary antibody in PBS with 1% BSA. Unbound secondary antibody on the sections was washed away with PBS twice, and the tissues were then stained with DAPI for 5 min, and finally mounted with ProLong Gold (ThermoFisher Scientific). Primary antibodies were mouse anti-filaggrin (Santa Cruz Biotech # sc-66192), rabbit anti-loricrin (Biolegend, # 905104), and rabbit anti-KI67 (Novus, # NB500-170). Secondary antibodies were goat anti-rabbit Alexa fluor 594 (Thermo Fisher, R37117) and goat anti-mouse Alexa fluor 488 (Thermo Fisher, A-11001).

### Tissue imaging and image analysis

H&E sections were imaged using a Motic EF-N Plan 10× objective with a 0.25 numerical aperture on a Motic BA210 microscope equipped with an EOS Rebel SLI camera controlled by Canon EOS software. Auto brightness and background adjustments were made to the images. Epidermal thickness was assessed in H and E sections by measuring the nucleated epidermal region of 10 or more randomly selected regions per section from 2 images, and 1 section per tissue. The average thickness is plotted from 5 tissues per treatment condition. An ordinary one-way ANOVA followed by Dunnett’s multiple comparisons test was used to compare differences from the axenic control.

Fluorescently stained tissues were imaged using a 10× objective on a Leica SPE confocal microscope controlled by Leica Application Suite X. The Leica Application Suite X was also used to generate mosaic images from six fields of view and maximum-intensity projections of z-stacks. Image J1.52s was used to count the number of KI67^+^, filaggrin^+^, and loricrin^+^ cells. In each case, maximum-intensity projections were smoothened and then binary images were generated based on a constant fluorescence intensity threshold. The ‘analyze particles’ tool was used to count the number of cells, limiting for circularity from 0.5 to 1 and a size cut-off of 30,000 [58].

## Results

### Microbiome representatives alter skin tissue gene expression

**Fig. 2:**
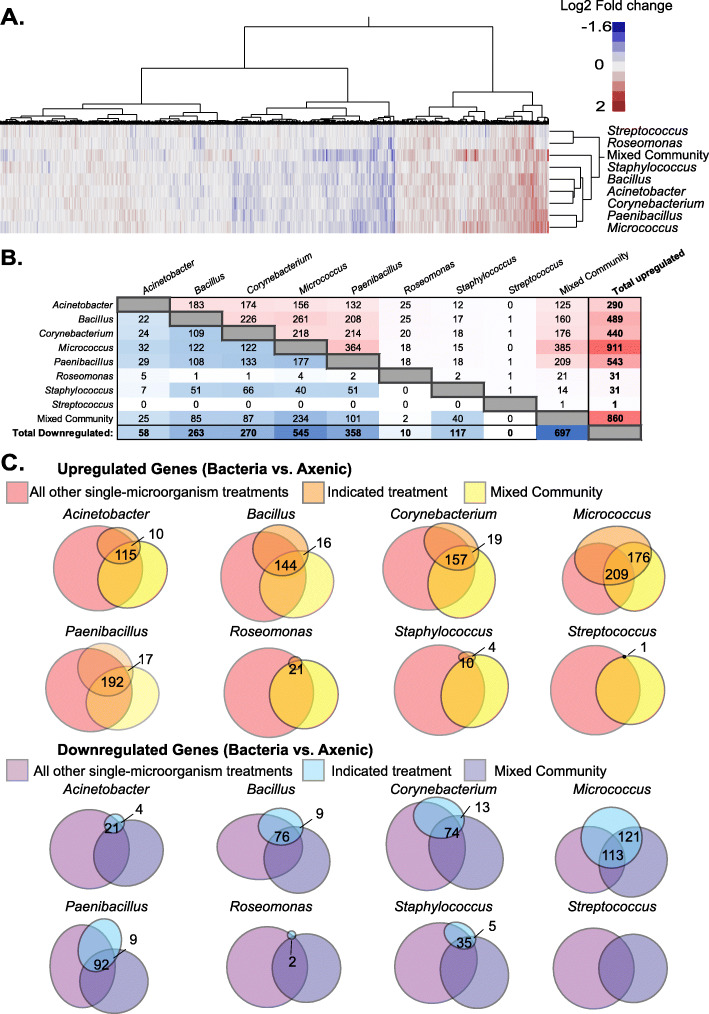
Comparison of differentially expressed genes across treatment groups. **a** Heat map showing log2 fold change of gene expression for microbiome treatments groups compared to the axenic control. **b** The number of shared differentially expressed genes between treatment groups. Upregulated genes are on the top right of the plot and downregulated genes are shown on the bottom left. **c** Euler diagrams comparing the number of differentially expressed genes in the indicated treatment conditions to all other single-microorganism treatments and the mixed community. Significantly altered gene expression was based on an adjusted *p* value < 0.05

To examine the impact of individual microorganisms on the skin tissue processes, model microbiome treatments were co-cultured at the air-tissue interface of EpiDerm skin tissue equivalents (see Fig. 1a). The inoculation density of each treatment condition was standardized to 5 × 10^5^ CFU per tissue. After 18 h of co-culture, entire tissues were subjected to transcriptomic analysis. We selected this shorter incubation, compared to the 5-day co-culture for histological analysis, to enable comparison of EpiDerm tissues with more standard compositions. As we examined the entire tissue, the transcriptomic analysis captured mammalian cell responses from both dermal fibroblasts and epidermal keratinocytes. To examine the degree to which individual treatment conditions led to distinct changes in gene expression, we performed a hierarchical clustering analysis on data from biological replicates of each treatment condition (see Fig. 1b). While the axenic treatment conditions clustered together, many tissues treated with individual microorganisms are interspersed and are not organized into clear and discrete clusters, suggesting that individual microorganisms elicit some overlapping responses in the host skin tissue. However, tissues treated with the mixed community clustered together more closely suggesting that they elicit a unique response that is distinct from the individual treatments. Indeed, the volcano plots in Fig. 1c show that some microbiome treatments elicit more pronounced alterations in gene expression (e.g., *Micrococcus luteus*) than others (e.g., *Streptococcus luteciae).* Interestingly, we see that tissues treated with *Staphylococcus epidermidis* had a greater bias towards downregulated, as opposed to upregulated, genes compared to the other conditions. In fact, this is the only condition where more genes were significantly downregulated than upregulated. To visualize relationships between each biological treatment group, a heat map of altered gene expression for the entire transcriptome is shown in Fig. 2a. Hierarchical clustering groups the *Streptococcus luteciae* and *Roseomonas mucosa* treatments, which elicited the least pronounced responses. Notably, the mixed community treatment is distinct from the remaining single-microorganism treatments.

### Microbiome representatives lead to distinct alterations in tissue gene expression

To examine how similar tissue responses were to each microorganism treatment, we compared how many differentially expressed genes (those with an adjusted *p* value < 0.05) were shared across treatment groups (Fig. 2b). Interestingly, we see that each treatment group elicits distinct changes in gene expression. For example, the *Staphylococcus epidermidis* and *Roseomonas mucosa* treatments both led to the upregulation of 31 genes, but the downregulation of 117 and 10 genes, respectively. They only share two upregulated genes in common (RNY5 and CPT1A) and do not share a single downregulated gene. *Acinetobacter lwoffi* and *Corynebacterium sp.* treatments are also clustered together in the hierarchical analysis (Fig. 2a), yet the differentially expressed genes elicited by each treatment have only partial overlap. *Corynebacterium* sp. and *Acinetobacter lwoffi* treatments led to the upregulation of 174 common genes, comprising 60% of those upregulated by *Acinetobacter lwoffi* treatment and 40% by *Corynebacterium* sp. treatment. They elicited a common 24 downregulated genes, 41% of the 58 downregulated by *Acinetobacter lwoffi* treatment and approximately 9% of the 270 elicited by *Corynebacterium* sp. treatment. Indeed, each microorganism leads to distinctive responses from the host skin tissue.

We next examined how unique the response of each single-microorganism treatment response was, relative to all other single-microorganism treatments. Additionally, we compared how much each single-microorganism treatment informed the response to the mixed community. Euler diagrams (shown in Fig. 2c) visualize these comparisons. Compared to all other conditions, six of the eight single-microorganism treatments led to the differential expression of unique genes. It is also interesting to see that a portion of genes up and downregulated by the mixed community treatment were not altered in any single-microorganism treatment. The portion of genes unique to the mixed community treatment underlies the importance of studying individual taxa in the presence of their microbiome community.

Bacterial activity may also play a role in differential skin tissue responses. We examined the RNA sequencing data for the presence of bacterial RNAs as an indicator of bacteria metabolic activity and viability (Figure S3).This data verifies that nearly all bacteria examined were transcriptionally active, including *Streptococcus luteciae*—a bacteria that elicited minimal responses from mammalian tissue. We also found that the largest number of bacterial RNA content in the mixed community condition, suggesting that the strong mammalian response is due, in part to bacterial activity. These reads were mostly attributed to *Streptococcus*, *Staphylococcus*, *Paenibacillus*, and *Micrococcus.*

### Mixed community treatment leads to the overrepresentation of genes involved in a variety of biological processes

**Fig. 3:**
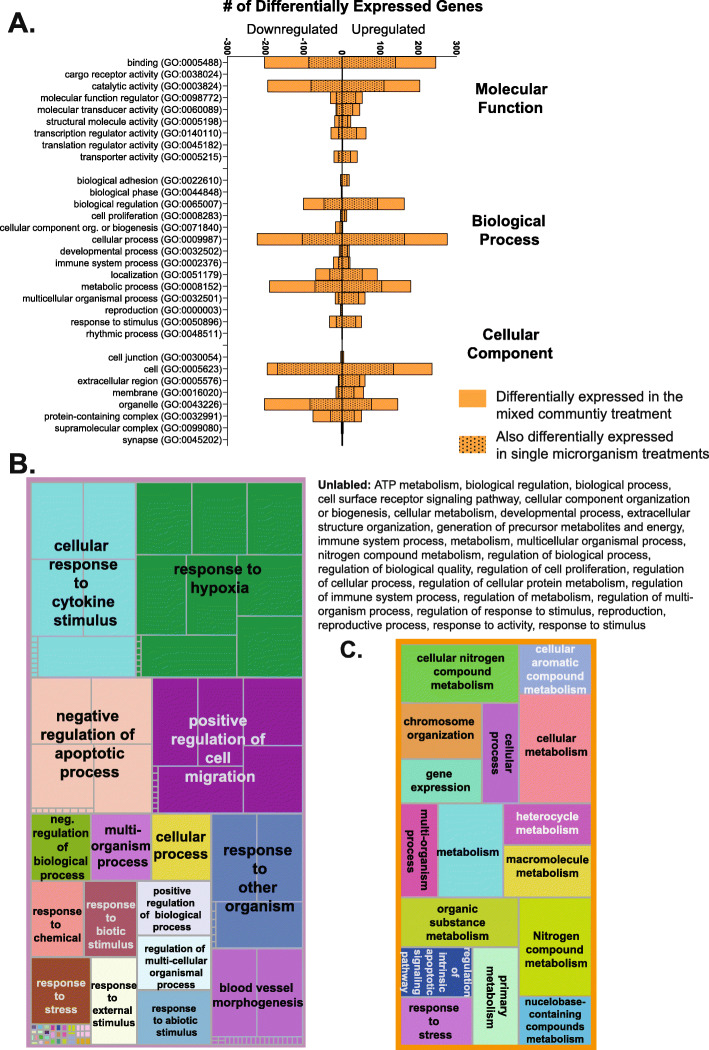
Gene sets overrepresented in mixed community treatment. **a** PANTHER GO-Slim functional classification of differentially expressed genes in the mixed community and the single-microorganism treatment. **b** Treemap showing enriched biological process gene sets for genes differentially expressed between the mixed community and axenic tissue. **c** Treemap showing enriched biological process gene sets for the genes which, compared to the axenic tissue, were differentially expressed in the mixed community but not differentially expressed in any single microorganism treatment. For treemaps, box size is indicative of enriched gene set significance. Genes with an adjusted *p* value < 0.05 were included in analyses

To better understand the alterations in gene expression, we classified differentially expressed genes were based on the high-level PANTHER GO-slim gene list (Fig. 3a). Genes that were altered in the mixed community but not altered in any single microorganism treatment were also classified and are shown in Fig. 3a. Differentially regulated genes have primary molecular functions of binding and catalytic activity, and many of those genes were unique to the mixed community treatment. In biological process gene sets, many genes are involved in metabolic processes, cellular processes, and localization. Genes are active inside the cell, organelles, protein-containing complexes, and the extracellular region.

To more specifically examine the processes influenced by the model microbiome treatment, we conducted a gene set overrepresentation analysis. We first examined gene sets that were overrepresented in the mixed community treatment compared to the axenic control. Over 200 gene sets were significantly enriched by mixed community treatment, and a full list of these gene sets is shown in Table S5. Many of these gene sets are redundant or have relational groupings. Thus, REVIGO [57], a freely available online tool, was used to summarize and better visualize the analysis. REVIGO uses a clustering algorithm to find a representative subset of GO terms and generate clusters of similar terms. This is shown as a treemap in Fig. 3b. Each colored box in the treemap shows clustered terms, with gray demarcations indicating overrepresented gene sets. The box sizing is reflective of the significance of overrepresented gene sets. A variety of biological processes were regulated, such as multicellular organism development, cell proliferation, regulation of apoptotic processes, and extracellular structure organization.

Next, to understand how the mixed community elicits unique responses to single-microorganism treatments, an additional gene overrepresentation analysis was conducted for genes differentially expressed in the mixed community treatment but not in any single-microorganism treatments (Fig. 3c). Numerous gene sets were identified, and a majority (10 out of 16) were involved in metabolism.

### Microbiome treatment influences epidermal thickness and cell proliferation in 3D skin tissue

**Fig. 4:**
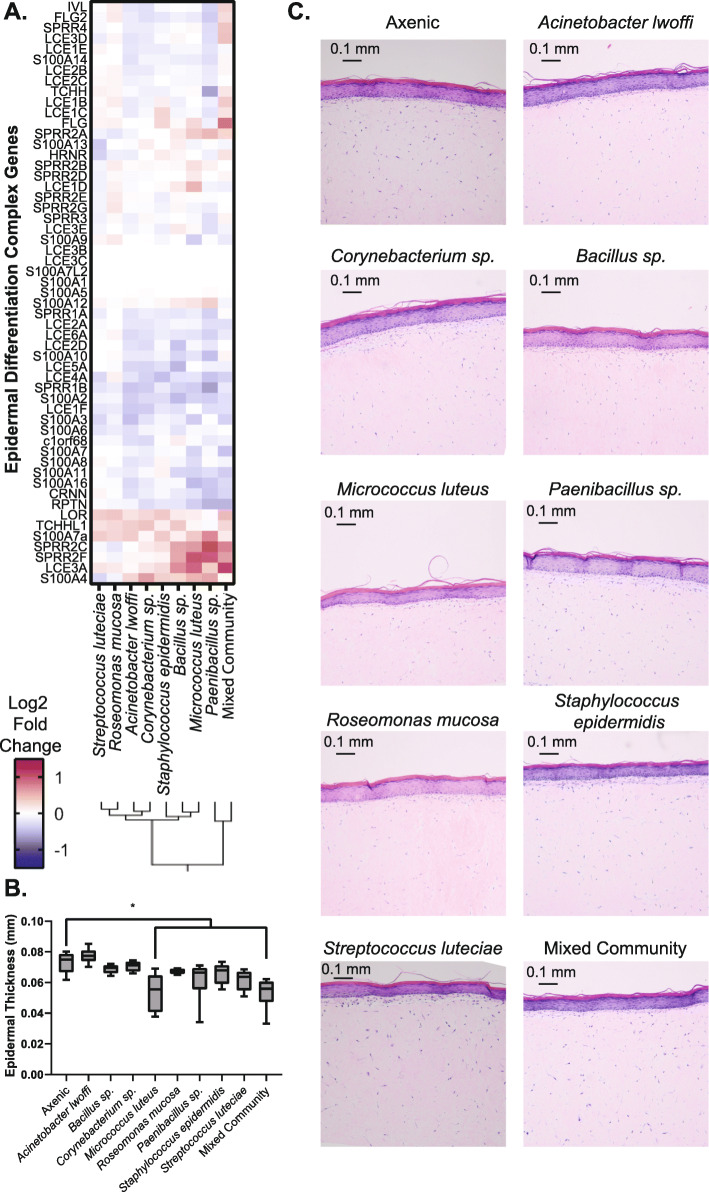
Microbiome treatments influence expression of epidermal differentiation genes. **a** Heat map showing the expression of genes involved in the epidermal differentiation complex for each microbiome treatment. **b** Measurement of thickness of the nucleated epidermal region across five tissues. * indicates condition is different from the axenic control, Dunnett’s test, *p* < 0.05. **c** Representative H&E images of tissues with each treatment condition

After broadly analyzing gene expression changes in response to microbe treatments, we then proceeded to characterize specific functions in more detail. First, to focus on genes involved in the differentiation and cornification of keratinocytes, we examined the expression of genes in the epidermal differentiation complex (see Fig. 4a). Similar alterations in gene expression are observed across the treatment conditions. Qualitatively, the most prominent alterations are elicited in the mixed community treatment. Next, skin tissues co-cultured with microbiome treatments for 5 days were stained with H&E and examined with microscopy. This longer co-culture incubation was selected to allow for the accumulation of functional changes within the tissue. The thickness of the nucleated epidermal region was measured and is plotted in Fig. 4b. Tissues treated with either the mixed community or *Micrococcus luteus* exhibit significantly reduced thickness. Representative images from each treatment condition are shown in Fig. 4c. No other prominent alterations in tissue structure were observed.

**Fig. 5.**
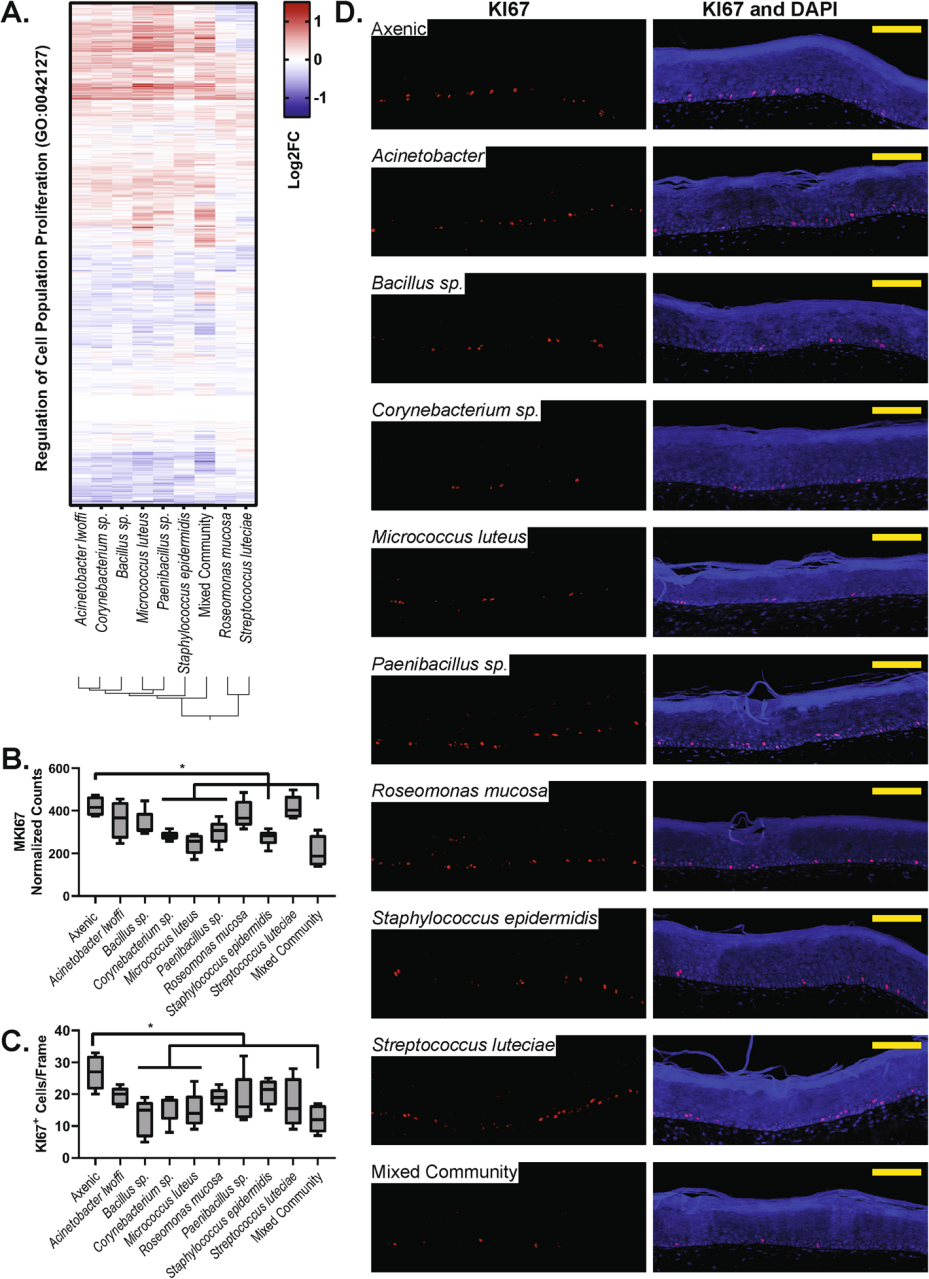
Microbiome treatment affects cell proliferation. **a** Heat map showing the expression of genes in the ‘Regulation of cell proliferation’ gene set. **b** Normalized counts of the *MIK67* transcript in each treatment condition. **c** The number of KI67^+^ cells counted in each treatment condition, across five tissues. * indicates condition is different from the axenic control, one-way ANOVA followed by Dunnett’s multiple comparisons test against the axenic control, *p* < 0.05. **d** Representative immunofluorescence images of each treatment conditions showing DAPI-stained nuclei in blue and KI67 in red. All scale bars indicate 100 μm

After examining cell differentiation, we then focused on cell proliferation. The expression of genes involved in the regulation of cell proliferation for all treatments is shown in Fig. 5a. Again, we observe a similar pattern in gene expression across treatments, with a reduced effect observed in *Roseomonas mucosa* and *Streptococcus luteciae* treatments. Next, we examined the differential expression of the cell proliferation marker *MKI67* (Fig. 5b). Mixed community treatment and four of the single-microorganism treatments (*Corynebacterium*, *Micrococcus*, *Paenibacillus*, and *Staphylococcus*) lead to downregulation of *MKI67*. To expand upon these findings, we stained tissue sections for Ki-67 expression and counted the number of Ki-67^+^ cells. Again, tissues treated with the mixed community as well as *Corynebacterium* sp., and *Micrococcus luteus*, had reduced numbers of proliferating cells (Fig. 5c). Representative images are shown in Fig. 5d. The proliferating cells are largely restricted to the basement of the epidermal region.

### Microbiome treatment influences the expression of key epidermal proteins

**Fig. 6:**
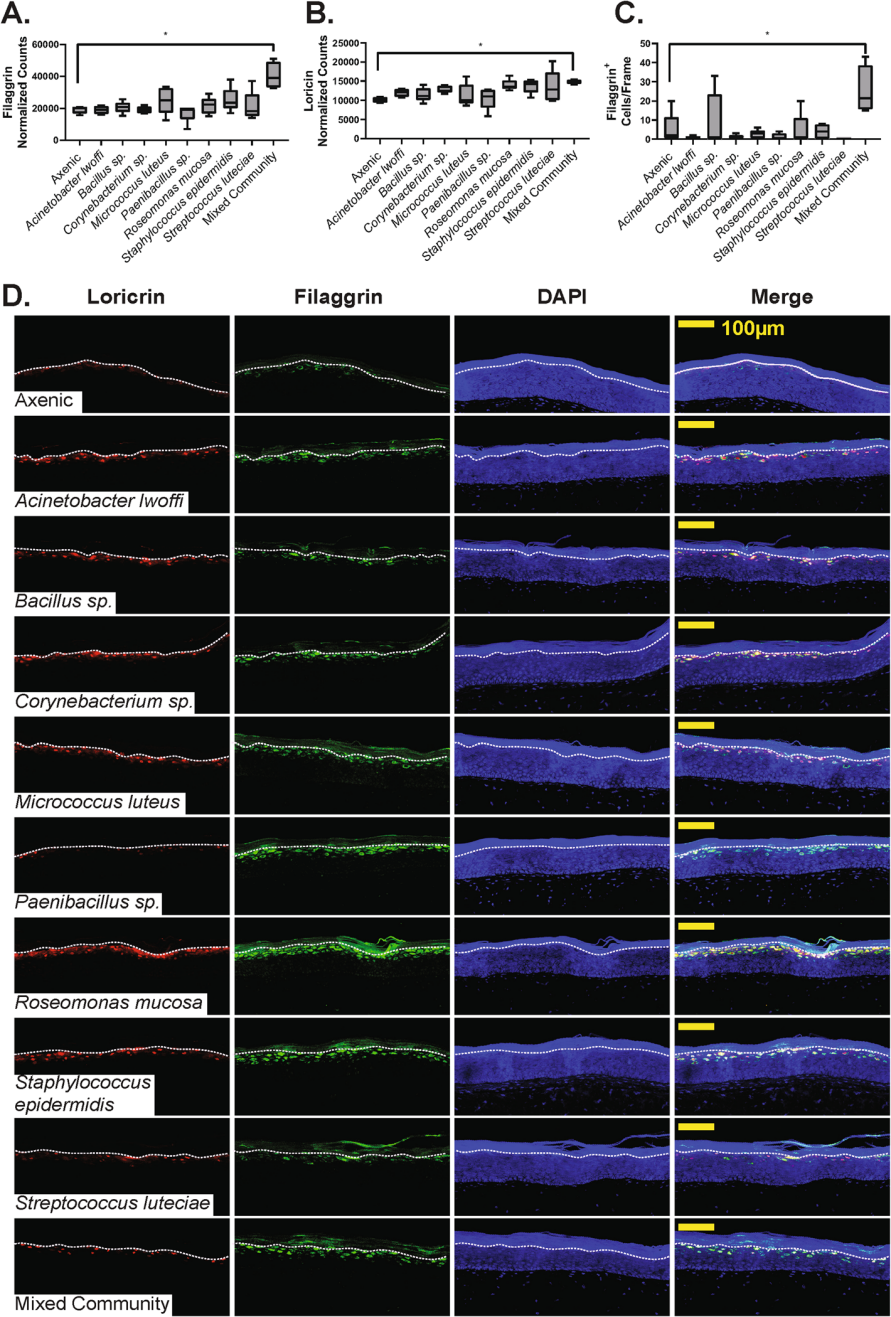
Microbiome treatment affects filaggrin and loricrin expression*.* Normalized counts of filaggrin (**a**) and loricrin (**b**) transcripts across treatment conditions. **c** The number of filaggrin^+^ cells counted in each treatment condition, across five tissues. * indicates condition is different from the axenic control, one-way ANOVA followed by Dunnett’s multiple comparison’s test against the axenic control, *p* < 0.05. All scale bars indicate 100 μm

Our examination of epidermal differentiation complex genes (Fig. 4a) also showed that filaggrin and loricrin—two genes important for skin barrier properties and skin structure—were impacted by microbiome treatment. Expression levels for filaggrin and loricrin are shown in Fig. 6a, b, respectively. Interestingly, only mixed-community treatment led to significant alterations in gene expression. However, the trend of loricrin upregulation was observed across many treatment conditions. To extend this finding to functional changes in skin tissue, we examined filaggrin and loricrin protein content in tissue sections. The number of cells staining positively for filaggrin content was quantified (Fig. 6c). Only the mixed community treatment leads to an increased presence of filaggrin. We also examined the number of filaggrin cells and their intensity, but did not see significant alterations. Representative images are shown in Fig. 6d and changes in filaggrin and loricrin expression are observed qualitatively.

We also observed that the expression of some AMPs were regulated by microbiome treatments (see Supplemental File S6). The AMP peptidoglycan recognition protein 2 (PGLYRP2) was significantly upregulated by multiple conditions—*Bacillus* sp., *Micrococcus luteus*, *Staphylococcus epidermidis*, and the mixed community. PGLYRP2 has amidase activity against bacterial peptidoglycan as well as bactericidal properties. Other studies have also observed its upregulation in skin tissue as a response to bacteria or cytokines [59, 60]. Additionally, we observed significant, but less prominent increases in neutrophil gelatinase-associated lipocalin (LCN2) in the *Bacillus* sp. treatment condition as well as beta-defensin 4A (DEFB4A) and protein S100-A7A (S100A7A) in the *Paenibacillus* sp. treatment condition.

## Discussion

Microbiome systems modulate a range of biological processes important in health and disease. Here, we investigated how representative members of the human skin microbiome influence cutaneous processes. In accord with previous studies in mice [33], we found that the skin microbiome regulates expression of genes important for epidermal differentiation and homeostasis. Using 3D skin tissue cultures, we observed that a model microbiome led to pronounced changes in epidermal thickness, epidermal cell proliferation, and filaggrin production. We additionally examined how individual microbiome constituents inform cutaneous responses to a diverse model microbiome. We found that no single organism drove host responses to the model microbiome community. These findings provide a basis for further investigation into the microbiome-skin dialogue.

Single microorganism treatments elicited similar but distinct responses in gene expression from skin tissue. Among the individual microorganism treatments, *Micrococcus luteus* elicited the strongest response. Additionally, *Micrococcus luteus* was the only individual microorganism treatment that led to a significant reduction in epidermal thickness, and one of the few treatments that reduced epidermal cell proliferation. This relatively robust response suggests that *Micrococcus luteus* may play a larger role in host-microbiome dialogue than previously anticipated.

In contrast, *Streptococcus luteciae* led to a nearly imperceptible response from skin tissue. Only one gene was significantly upregulated—Ro60-Associated Y5 (RNY5). This gene is one of several noncoding RNAs that bind to and regulate Ro60—an RNA binding protein; although the function of Ro60 and RNY5 are not well understood [61]. However, RNY5 is known to be upregulated in response to stress and is enriched in exosomes, suggesting it may play a role in cell-to-cell signaling [62, 63]. This gene was also upregulated in many other examined treatments (all except for *Acinetobacter lwoffi* and *Micrococcus luteus*), suggesting that it may be a conserved cellular response to microbiome presence. While microorganism treatments were standardized by colony-forming units upon administration, it is possible that *Streptococcus luteciae* grew very slowly or had lower metabolic activity—thus, eliciting a minimal tissue response. However, examination of bacterial reads in the RNA sequencing data identified *Streptococcus* RNAs, indicating its viability. The minimal skin responses could be attributed to slower growth rates or a lack of interaction with the skin tissue.

*Staphylococcus epidermidis* also elicited a distinct gene expression profile. Compared to other conditions, the response to *S. epidermidis* elicited a bias toward the downregulation, rather than the upregulation of genes. A previous microarray-based study of skin equivalent responses to *Staphylococcus epidermidis* similarly found a greater bias towards gene downregulation [64]. As one of the most abundant and prevalent species found on the skin, this suggests that the microorganism may have more unique interactions with host tissue.

We also examined a mixed community treatment consisting of each of the examined single microorganisms mixed in equal proportions. We found that this model microbiome led to significant alterations in cutaneous biological processes—evident through both transcriptomics and histological analyses. Gene expression was altered for a range of biological processes, including the immune response, epidermal differentiation, cell proliferation, regulation of apoptosis, and metabolism. These transcriptional profiles manifested in alterations to epidermal thickness, cell proliferation, and filaggrin protein content. Changes in epidermal thickness and cell proliferation were also only observed in a few of the individual microorganism treatments. Significant increases in loricrin and filaggrin gene transcripts and filaggrin protein content were only observed for tissues treated with the mixed community, and not for any individual microorganism treatments. We also observed that host responses to the mixed community were not entirely driven by any single microorganism, highlighting the unique properties of a diverse microbiome community in host-microbiome interactions. Collectively, these results suggest a community effect in microbiome-host signaling.

These findings for the mixed community are largely in accord with research investigating the influence of the skin microbiome using germ-free mice. Specifically, Meisel et al. used transcriptomics to show that the microbiome impacts gene expression involved in epidermal barrier formation and differentiation [33]. However, gene set enrichment analysis in this study found that immune response genes were more significantly altered than in our study. This could potentially be a result of in vivo-in vitro differences of cell types present, as Meisel et al. found that a variety of immune cells had increased production of IL-1ɑ and IL-1β within the skin, and these cells were not included in our 3D tissue model. Additionally, the model microbiome community used in our study was more simplistic than communities found on mammalian skin. Bacteria strains that were not included in this study could lead to stronger immune responses.

This study examined a single seeding density of bacteria on skin tissue, a control enabled by using an in vitro tissue culture system. However, it is important to note that bacterial strains likely grow at different rates, which could influence their interactions with host tissue. These variations in growth rates could influence comparisons between single-microorganism and mixed-community treatments. Follow-up studies could examine the impact of bacterial density, mixed community compositions, and growth rates on host-microbiome interactions.

For our investigations of skin-microbiome interactions, we employed full-thickness human skin tissue models. These models include dermal fibroblasts and epidermal keratinocytes, enabling paracrine signaling between the cell types, which is known to influence processes such as keratinocyte proliferation and differentiation [65]. It is important to note that studies indicate heterogeneity can exist across tissue models arising from the source of cells and the culturing medium and matrix substrates used for tissue development [66, 67]. As tissues used in our study are commercially available, other scientific groups should be better enabled to expand upon the presented work. The commercial model that we have chosen satisfies the majority of parameters recently outlined for skin models intended for barrier function research [68]. A general limitation of current skin tissue models is the lack of sebaceous glands and hair follicles. This prevents co-culturing *Cutibacterium*, a highly prevalent and abundant bacteria of the skin microbiome that primarily resides within the anaerobic environment of sebaceous glands. Future development of more sophisticated models with sebaceous glands may facilitate *Cutibacterium* studies, and we anticipate these developments will be highly advantageous to skin microbiome research.

While the use of in vitro tissue culture systems enabled controlled studies, the cell types were sourced from single donors. Future work could focus on the variability of responses to the skin microbiome that occur across the human population. For example, age is associated with changes in microbiome composition [69], skin properties [70], and transcriptome activity [71, 72]. Additionally, African and Caucasian skin types have been found to have different protein expression for filaggrin processing, epidermal morphogenesis, and differentiation [73]. Future studies could identify cutaneous responses to the microbiome that are consistent across the variable human population.

Our findings support that skin microbiome constituents elicit varied cutaneous biological responses. There still exist significant gaps in understanding the mechanisms that drive these varied responses, especially among commensal microorganisms. This study provides a basis for investigating these mechanisms. For example, since we examined the transcriptomic responses from whole tissues—including both epidermal and dermal cells—the contributions and interactions between fibroblasts and keratinocytes remain to be elucidated. Additionally, follow-up studies could examine the extent that individual species or strains of microorganisms elicit varied biological responses. For example, if it is common for *Streptococcus* bacteria to elicit minimal responses from skin tissue. An interesting finding was how individual microorganism treatments did not predict responses to the mixed community treatment. Therefore, bacterial transcriptomics or metabolomics could elucidate how microbial activity may change in the presence of a mixed community.

## Conclusions

Our efforts reveal the effects of several prominent skin microbial taxa on human skin tissue and point to a pronounced community-effect on the host skin that cannot be attributed to any single taxa acting alone. This community-effect entails not only a distinct signature in the host transcriptional response profile, but also on epidermal thickness, cell proliferation, and filaggrin and loricrin observations. This work and other studies make it clear that individual microorganisms can elicit distinct responses from host tissue. However, we additionally find that host responses to individual bacteria are not fully predictive of the responses to a mixed community. We recommend that future studies examining microorganism-host relationships characterize the impact of specific microorganisms in a mixed community so that responses unique to the community context are captured. We envision that this work, along with additional efforts to elucidate the mechanistic underpinnings of community-induced host-microbiome interactions, will inform therapeutic applications of probiotic development, particularly in the realms of synthetic biology and microbiome engineering.

## Supplementary Information

**Additional file 1.** Microorganism Isolate Information. Table containing information for the bacterial microorganisms used in this study. Table containing the identifying genus, the isolate identifier used by the collecting study, the 16S rRNA sequence, and the least common ancestors of the bacterial isolate determined by classification to the Greengenes, RDP, and SILVA databases [42].

**Additional file 2: Figure S1.** Optical Density and Colony Forming Unit Standardization. Plots showing relationships between the colony forming units per milliliter and optical density at 600 nm of cultures for each bacterial isolate. **Figure S2.** Distribution of bacteria representatives on EpiDerm following overnight incubation. An equivalent colony-forming unit mixture of *Staphylococcus aureus* and P*seudomonas aeruginosa* totaling 5X10^5^ bacteria were administered to the air-tissue interface of EpiDerm and incubated overnight. A half hour prior to microscopy, EpiDerm tissue media treated with NucBlue Live reagent from the Blue/Green ReadyProbes™ Cell Viability Imaging Kit (Invitrogen). The tissue was then inverted on a glass coverslip and imaged using confocal microscopy. Extended focus images are shown. **Figure S3.** Microbial classifications on EpiDerm 18 hours after treatment (A) Number of reads for the target genus found in each of the specified conditions. Reads from all taxa of interested are summed together for the axenic and mixed community conditions. (B) Reads for target taxa are shown across all treatment groups. Values are expressed as a fraction of the number of reads found for the target taxa in the monoculture treatment conditions. (C) Composition of microbial taxa found in the Mixed Community Treatment Condition.

**Additional file 3.** Quality Assessment of RNA-Sequencing Data. This file shows quality control metrics for the sequences of each individual sample. This includes the number of reads in the sample, the number and percentage of those reads that were mapped to the transcriptome, the number and percentage of total transcripts covered, and the number and percentage of total genes hit. Additionally, we assessed Pearson correlations of each individual sample within a biological treatment group.

**Additional file 4.** Differential Gene Expression Data. This file contains the differential gene expression analysis for each microbiome treatment relative to the axenic control. Each gene included in the analysis is shown, including the basemean, log2 fold change, log2 fold change unshrunk, p-value, and adjusted p-value, as given by DESeq2.

**Additional file 5.** Gene Set Overrepresentation Test Data. This file contains input and output information important to the Gene Set Overrepresentation Test. Genes used for the background gene list. The output data from the PANTHER Overrepresentation tests is also shown. (PDF 1358 kb)

**Additional file 6.** Antimicrobial Peptide Gene Expression. This file contains differential gene expression data (Log2 fold change and the adjusted p-value) for genes that encode for antimicrobial proteins and peptides. Citations for the individual genes are also includes. (XLSX 250 kb)

## Data Availability

Raw transcriptomic data files analyzed in this study are available in the Sequence Read Archive at https://www.ncbi.nlm.nih.gov/sra/PRJNA606973 or by searching for reference PRJNA606973.
