# Supplementary material for: A mixed community of skin microbiome representatives influences cutaneous processes more than individual members
Source: Microbiome. 2021 Jan 22;9:22. doi: 10.1186/s40168-020-00963-1 (PMC7825201; doi:10.1186/s40168-020-00963-1)
Supplement: Supplementary file 3 — Additional file 2: Figure S1. Optical Density and Colony Forming Unit Standardization. Plots showing relationships between the colony forming units per milliliter and optical density at 600 nm of cultures for each bacterial isolate. Figure S2. Distribution of bacteria representatives on EpiDerm following overnight incubation. An equivalent colony-forming unit mixture of Staphylococcus aureus and Pseudomonas aeruginosa totaling 5X105 bacteria were administered to the air-tissue interface of EpiDerm and incubated overnight. A half hour prior to microscopy, EpiDerm tissue media treated with NucBlue Live reagent from the Blue/Green ReadyProbes™ Cell Viability Imaging Kit (Invitrogen). The tissue was then inverted on a glass coverslip and imaged using confocal microscopy. Extended focus images are shown. Figure S3. Microbial classifications on EpiDerm 18 hours after treatment (A) Number of reads for the target genus found in each of the specified conditions. Reads from all taxa of interested are summed together for the axenic and mixed community conditions. (B) Reads for target taxa are shown across all treatment groups. Values are expressed as a fraction of the number of reads found for the target taxa in the monoculture treatment conditions. (C) Composition of microbial taxa found in the Mixed Community Treatment Condition. [file 40168_2020_963_MOESM3_ESM.docx]

**Figure S1. Optical Density and Colony Forming Unit Standardization**

**
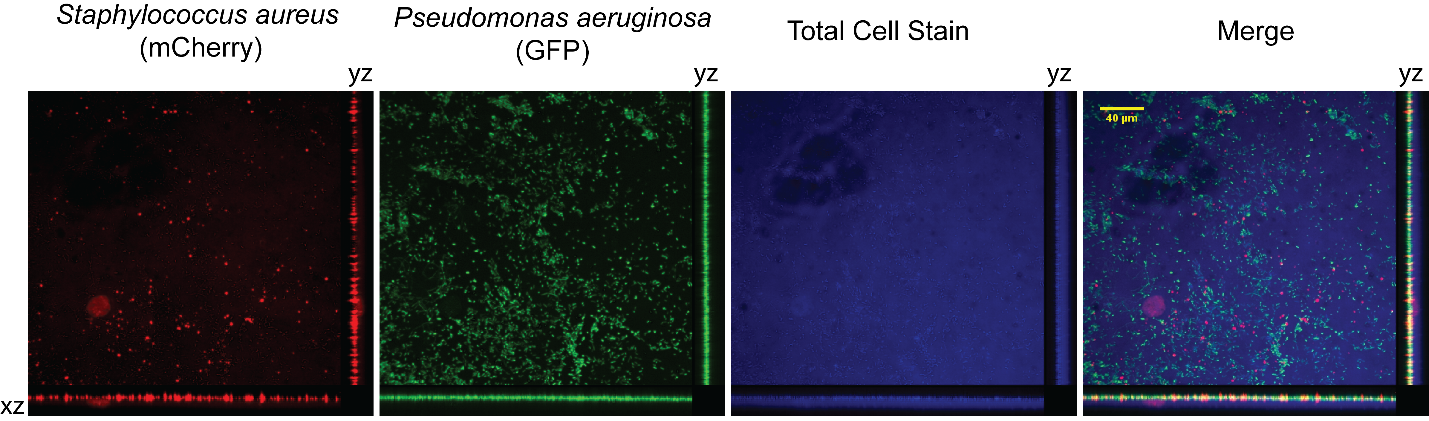
Figure S2. Distribution of bacteria representatives on EpiDerm following overnight incubation.**


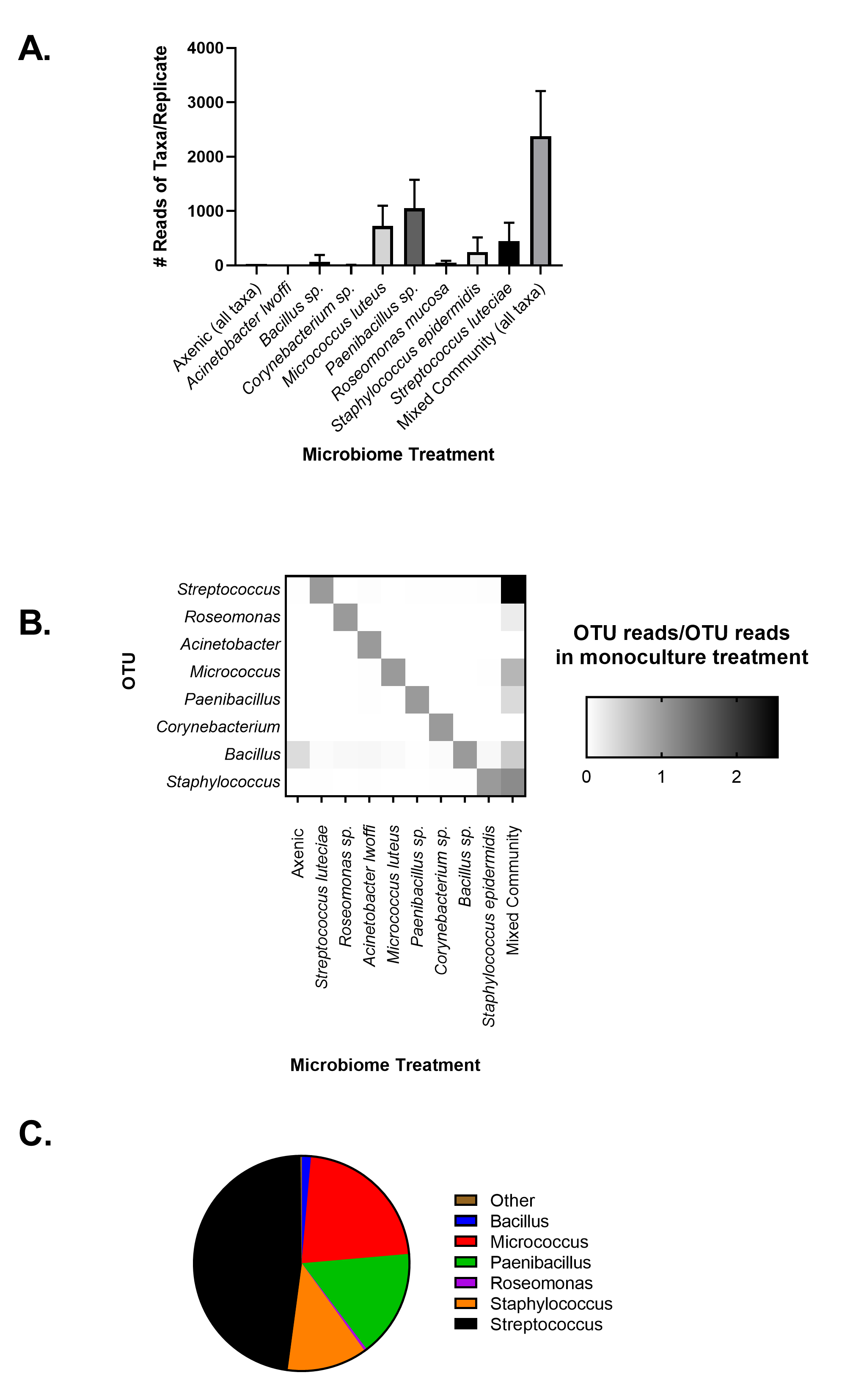


**Figure S3. Microbial classifications on EpiDerm 18 hours after treatment**
